# L-Arginine Exerts Excellent Anti-Stress Effects on Stress-Induced Shortened Lifespan, Cognitive Decline and Depression

**DOI:** 10.3390/ijms22020508

**Published:** 2021-01-06

**Authors:** Monira Pervin, Keiko Unno, Tomokazu Konishi, Yoriyuki Nakamura

**Affiliations:** 1 Tea Science Center, Graduate School of Integrated Pharmaceutical and Nutritional Sciences, University of Shizuoka, Shizuoka 422-8526, Japan; yori.naka222@u-shizuoka-ken.ac.jp; 2 Faculty of Bioresource Sciences, Akita Prefectural University, Shimoshinjo Nakano, Akita 010-0195, Japan; konishi@akita-pu.ac.jp

**Keywords:** aging, arginine, brain, chronic psychosocial stress, depression, oxidative damage, shortened lifespan

## Abstract

The anti-stress potential of dietary L-arginine (Arg) was assessed in psychosocially stress-loaded senescence-accelerated (SAMP10) mice. Although this strain of mouse is sensitive to stress, daily administration of Arg at 3 mg/kg significantly suppressed aging-related cognitive decline and behavioral depression at nine months of age and counteracted stress-induced shortened lifespan. To investigate the mechanism of the anti-stress effect of Arg in the brain, early changes in oxidative damage and gene expression levels were measured using SAMP10 mice that were stress-loaded for three days. Increased lipid peroxidation in the brains of stressed mice was significantly lowered by Arg intake. Several genes associated with oxidative stress response and neuronal excitotoxic cell death, including *Nr4a1*, *Arc*, and *Cyr61*, remarkably increased in response to psychosocial stress; however, their expression was significantly suppressed in mice that ingested Arg even under stress conditions. In contrast, the genes that maintain mitochondrial functions and neuronal survival, including *Hba-a2* and *Hbb-b2*, were significantly increased in mice that ingested Arg. These results indicate that Arg reduces oxidative damage and enhances mitochondrial functions in the brain. We suggest that the daily intake of Arg plays important roles in reducing stress-induced brain damage and slowing aging.

## 1. Introduction

We have conducted research on the anti-stress effect of theanine, an amino acid that is mainly found in the leaves of *Camellia sinensis* L. (green tea) [1,2,3,4,5,6,7,8]. In our previous studies wherein the anti-stress effects of other amino acids in green teas were examined, L-arginine (Arg) was found to exert an excellent anti-stress effect that is similar to or better than that exerted by theanine [4]. Almost the same anti-stress effect of Arg was observed at 0.032–3.2 mg/kg/day. Arg is the second most abundant amino acid, after theanine, in high-grade green teas [4]. Chronic psychosocial stress has been demonstrated to shorten lifespan and accelerate age-related alterations such as cerebral atrophy, oxidative damage, cognitive dysfunction, and behavioral depression in stress-loaded senescence-accelerated mouse prone 10 (SAMP10) [1,2]. Mice of this strain have been reported to exhibit a short lifespan, aging-related brain atrophy, and cognitive decline even under normal conditions [8,9,10,11] and are sensitive to stress [8]. Therefore, we aimed to further elucidate whether Arg has anti-stress potential against chronically-stressed SAMP10 mice in the present study. 

Fundamentally, Arg, one of the 20 basic natural amino acids, is functionally classified as an essential amino acid in birds, carnivores, and young mammals and semi-essential for adults, and has been identified to play critical roles in health, including immune response [12], wound healing [13], growth hormone release [14], and cell proliferation [15]. Dietary sources of Arg include meat, wheat, sea foods, milk, cheese, corn, soy, nuts, and others [16]. In Japanese green tea, Arg is contained in the range from 0.85 to 3.14 mg/g as a free amino acid [17]. In previous studies, dietary Arg has been reported to suppress oxidative stress [18] and inflammatory responses [19]. Ingested Arg via the gastrointestinal tract is absorbed in the small intestine. Thereafter, approximately 40% of the ingested Arg is circulated systemically [20]. Dietary Arg is degraded by arginase, which converts Arg into urea, ornithine, proline, polyamines, glutamate, and glutamine [21]. In addition, nitric oxide, which can be converted from Arg by nitric oxide synthase [21], acts as a precursor of signaling molecules [22] and is involved in several functions, including the vasodilation of blood vessels [21], synaptic plasticity [23], learning and memory processing [24], and modulation of neuronal function during stress and anxiety [25]. Therefore, Arg and its metabolites play many important roles in health. 

Chronic psychosocial stress has been associated with various mental disorders such as depression and anxiety [26]; it elevates the risk of neurodegenerative diseases, including Alzheimer’s disease, dementia [27], and cardiovascular diseases, accelerates aging, and shortens lifespan [28]. Numerous animal and human studies have shown the deleterious effects of stress on the brain, behavior, and cognitive function [1,8,29,30,31]. The brain is highly susceptible to stress during both early childhood and old age [32]. Stress activates the hypothalamic–pituitary–adrenal axis which leads to the secretion of glucocorticoids from the adrenal glands [32,33]. Increased levels of glucocorticoids have been associated with neuronal loss [34,35], cognitive impairment, and Alzheimer’s disease development [36].

In the present study, to elucidate the anti-stress potential of Arg on stress-loaded SAMP10 mice, the long-term effect of stress was observed by measuring the cognitive function and depressive-like behavior of mice at nine months of age. Furthermore, the lifespan of these mice was measured. Next, to elucidate the mechanism of Arg in the brain, the initial responses of SAMP10 mice loaded with stress for three days were used to observe the changes in lipid peroxidation (LPO) and gene expression levels in the hippocampus and prefrontal cortex.

## 2. Results

### 2.1. Long-Term Effect of Stress

#### 2.1.1. Improving Effect of Arg on Learning Ability and Behavioral Depression

**Figure 1:**
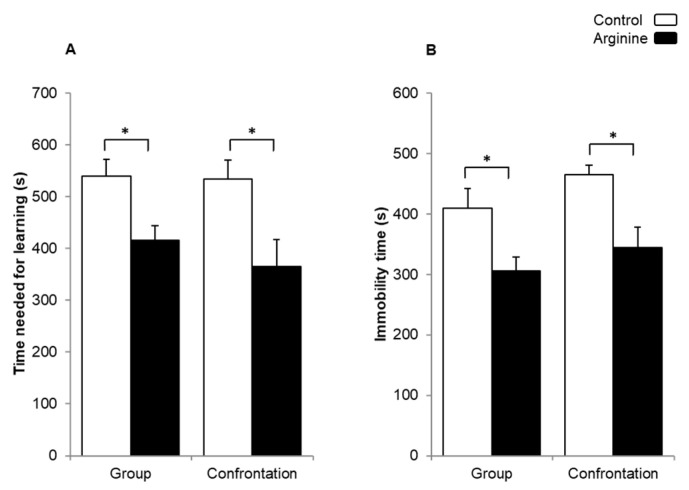
Effect of Arg intake on learning ability and behavioral depression in SAMP10 mice. The step-through passive avoidance test (**A**) and the tail suspension test (**B**) were performed using 9-month-old mice. Mice ingested Arg (3 mg/kg, closed column) or only water (control, open column) (the number of mice in each group = 12; * *p* < 0.05).

The learning ability of mice was measured at nine months of age using a step-through passive avoidance test. A longer learning time implies lower learning ability. The mice that ingested Arg under both confrontation and group housings showed a significantly shortened learning time than the control mice that consumed only water under both housing conditions (Figure 1A). The group-housed mice were defined as mice under a low-stress condition; however, the group-housed mice used in this experiment sometimes fought and might have been stressed similarly as the mice that were confrontationally housed. Therefore, no difference in terms of learning ability between the mice that were group-housed and confrontationally housed was observed in this experiment.

The effect of Arg intake on behavioral depression was investigated using a tail suspension test at nine months of age. The immobility duration was significantly shorter in the confrontationally housed and group-housed mice that ingested Arg than the control mice that were also confrontationally and group-housed (Figure 1B). 

#### 2.1.2. Improving Effect of Arg Intake on Lifespan

**Figure 2:**
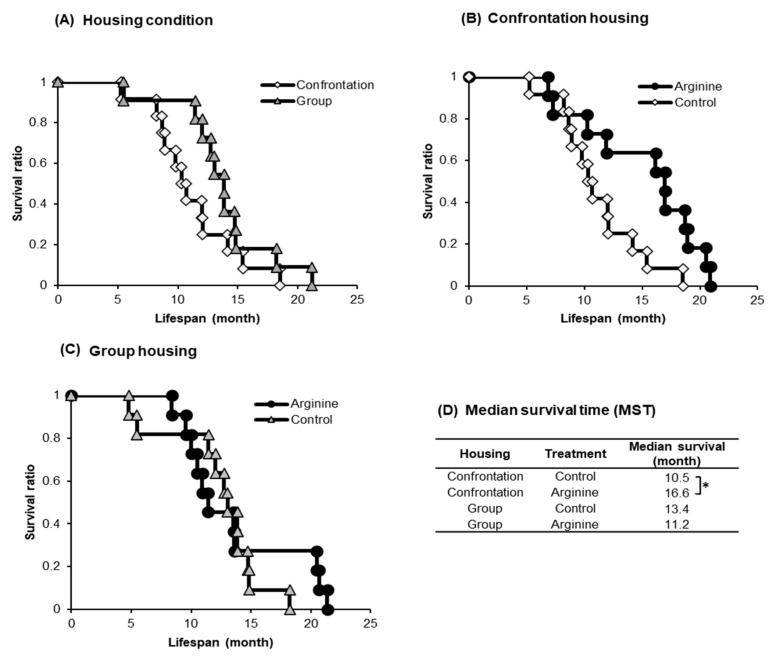
Effect of Arg intake on longevity in SAMP10 mice. Effect of housing condition (**A**), effect of Arg intake on confrontation housing (**B**), effect of Arg intake on group housing (**C**), and median survival time of each group (**D**). Four groups of mice, the number of mice in each group, *n* = 12 (6 mice were housed per cage for group housing and 2 mice per cage for confrontation housing). Mice ingested Arg at 3 mg/kg or only water (control) freely from 1 month of age. Arg-water (10 µg/mL) was freshly prepared twice a week. * *p* < 0.05.

Many among the confrontationally housed mice died earlier than the group-housed mice (Figure 2A). The median survival time (MST) of the confrontationally housed mice that consumed only water was 10.5 months, whereas that of the mice administered Arg at 3 mg/kg under confrontational housing was 16.6 months (Figure 2B,D); this MST was 1.58 times longer (*p* = 0.032) than the MST of confrontationally housed mice that consumed only water. In contrast, no difference in MST was observed in the group-housed mice by Arg intake (Figure 2C) (*p* = 0.75) and between confrontational and group housing control mice (Figure 2A) (*p* = 0.17).

### 2.2. Initial Response to Stress

#### 2.2.1. Oxidative Damage in the Brain

**Figure 3:**
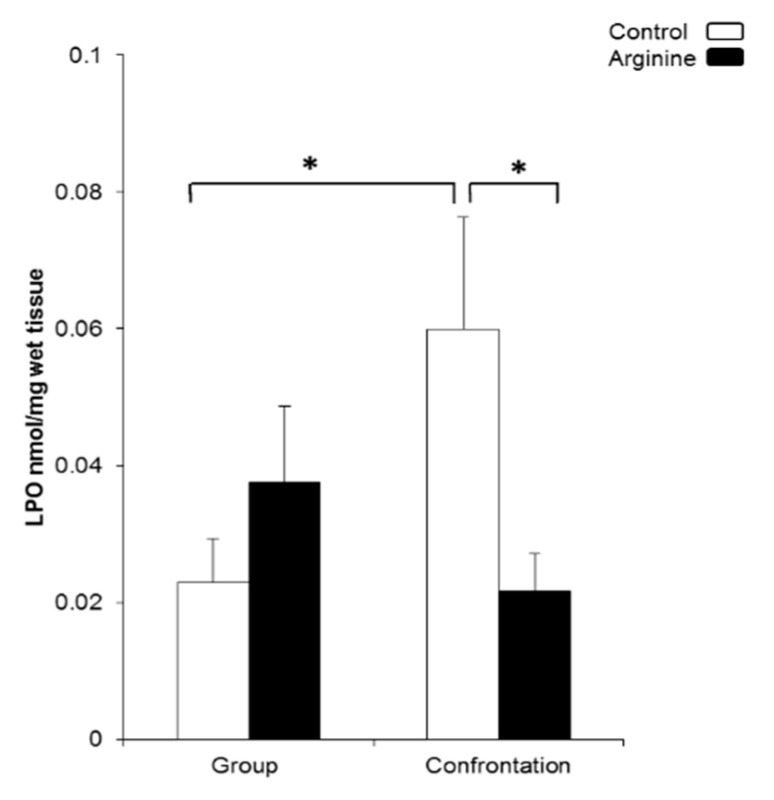
Levels of oxidative damage in the brain. Lipid peroxidation (LPO) in the brain was measured using the cerebral cortex. Mice were housed confrontationally for 3 days after single housing for 1 month. Mice ingested Arg (3 mg/kg, closed column) or only water (control, open column) (*n* = 5~6, * *p* < 0.05, Fisher’s least significant differences).

The LPO levels in the cerebral cortex, a marker for oxidative damage, were measured using the mice that were confrontationally housed for three days. Same-aged group-housed mice were used as a reference. LPO levels were significantly higher in the confrontationally housed control mice than those of the group-housed control mice and the mice that ingested Arg under confrontational housing (Figure 3). No difference in LPO levels due to Arg intake was observed in group-housed mice (Figure 3).

#### 2.2.2. Effect of Arg Intake on Gene Expression in the Hippocampus of Stressed SAMP10 Mice

**Figure 4:**
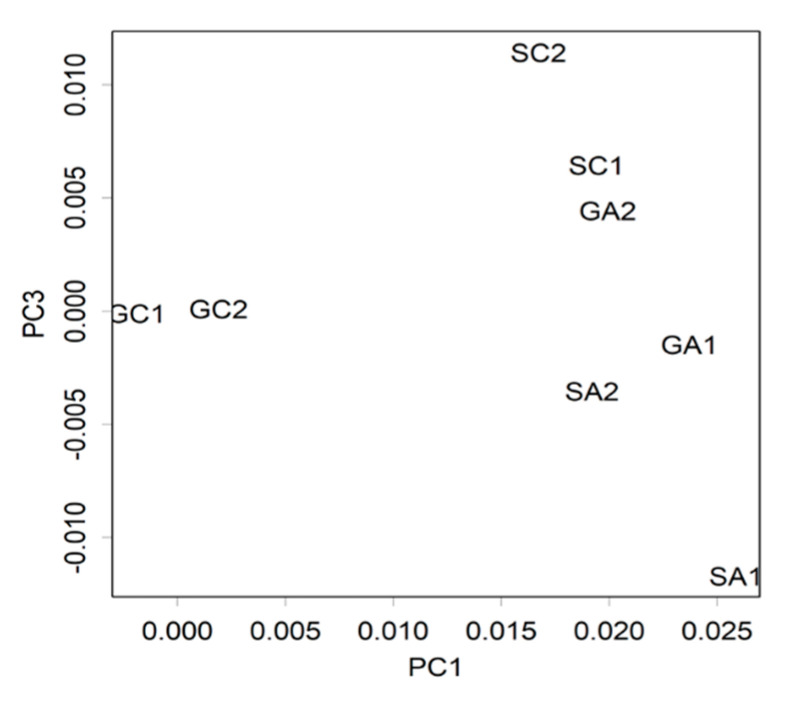
Principal component analysis of gene expression. Hippocampal samples were obtained from mice housed confrontationally for three days. Group-housed mice were kept in a cage for one month. The principal component (PC) ordination of ANOVA-positive genes is based on the transcriptome of hippocampal gene expression in mice of GC: group-control; GA: group-Arg; SC: confrontation stress-control, SA: confrontation stress-Arg (*n* = 2 for each group).

To investigate the mechanism of action of Arg in the brain, we performed microarray analysis to assess the comprehensive changes in gene expression using the hippocampus of mice. The early changes in gene expression on the third day of stress loading were examined. In the microarray analysis, all four groups of mice’s hippocampus tissues were used including group-control, group-Arg, confrontation-control, and confrontation-Arg. As a result of principal component analysis (PCA), changes due to Arg intake appeared along with the PC1 axis. Meanwhile, changes due to Arg intake under the stress condition coincided with the PC3 axis. The data are shown simultaneously in a biplot (Figure 4). The top 10 genes that were significantly downregulated and upregulated in the mice that ingested Arg are listed in Table 1. Among these genes, nuclear receptor subfamily 4, group A, member 1 (Nr4a1), also known as Nur77, was the most downregulated gene after Arg intake. Nr4a1 is a potent pro-apoptotic member of the nuclear receptor superfamily and is associated with neuronal excitotoxicity and neuronal cell death [37,38,39]. Activity-regulated cytoskeleton-associated protein (Arc) has been shown to induce neuronal cytotoxicity and cell death [40]. Membrane-spanning 4-domains, subfamily A, member 6 D (Ms4a6d) is a transmembrane protein involved in inflammatory signaling [41] and Alzheimer’s disease [42,43]. Moreover, midline 1 (Mid1) has been reportedly expressed in the brains of patients with Alzheimer’s disease [44]. Cysteine rich protein 6 (Cyr61) has been associated with neuronal cell death [45]. 

In contrast, melanoma antigen (Mela), whose function is unknown, is the most upregulated gene in the hippocampus of mice that ingested Arg. Hemoglobin is the iron-containing protein that carries oxygen in vertebrate erythrocytes. Hemoglobin α- and β-chains (Hba and Hbb) were found to be expressed in several brain regions, including the cortex and hippocampus of rats [46]. Furthermore, these proteins have been identified within the mitochondrion of neurons [47] and are important in maintaining mitochondrial functions and neuronal survival.

#### 2.2.3. Effect of Arg Intake on Nr4a1, Arc, and Cyr61 Levels in the Brain

**Figure 5:**
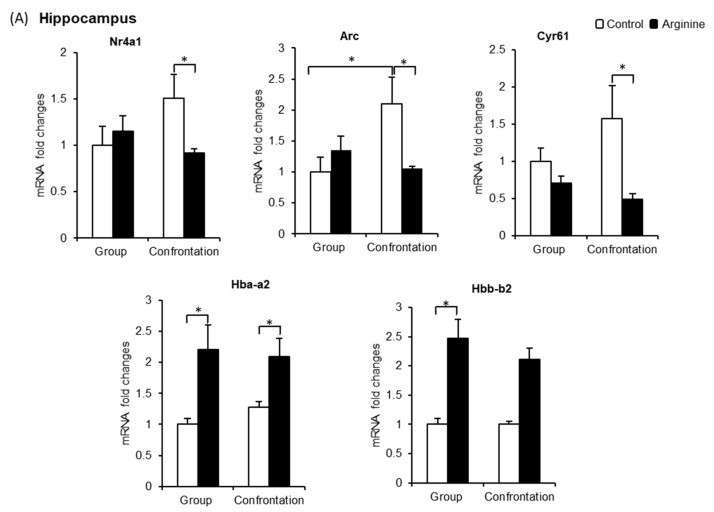

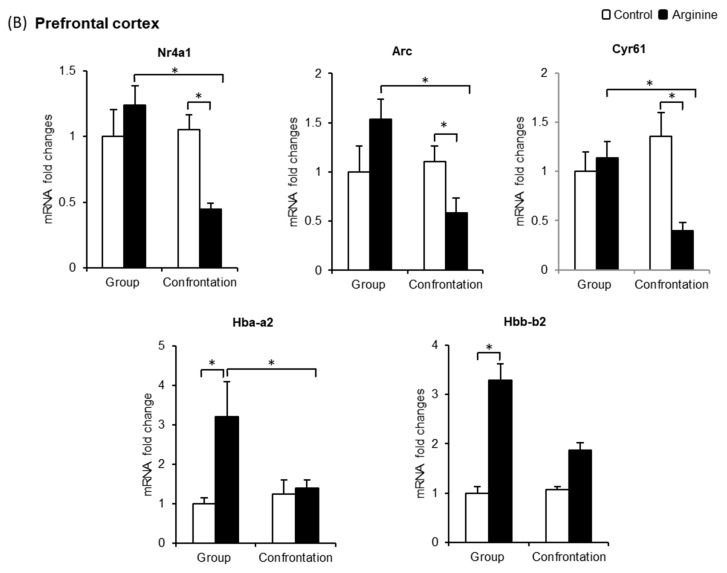
(**A**) Expression of *Nr4a1*, *Arc*, *Cyr61*, *Hba-a2*, and *Hbb-b2* in the hippocampus of mice under group and confrontation housing. Mice ingested Arg (3 mg/kg, closed column) or only water (control, open column) (*n* = 5~6, * *p* < 0.05, Fisher’s least significant differences). (**B**) Expression of *Nr4a1*, *Arc*, *Cyr61*, *Hba-a2*, and *Hbb-b2* in the prefrontal cortex of mice under group and confrontation housing. Mice ingested Arg (3 mg/kg, closed column) or only water (control, open column) (*n* = 5~6, * *p* < 0.05, Fisher’s least significant differences).

As the *Nr4a1*, *Arc*, and *Cyr61* genes are the most significantly downregulated genes after Arg intake (detected using microarray analysis), we focused on these genes and compared their expression levels in both the hippocampus and prefrontal cortex tissues of group-housed mice using quantitative real-time reverse transcription polymerase chain reaction (qRT-PCR) (Figure 5). Arg intake exerted no effect on these genes in group-housed mice; however, the gene expression levels of *Arc* in confrontationally housed mice were significantly higher than those of group-housed mice. Furthermore, the expression levels of *Nr4a1* and *Cyr61* in confrontationally housed mice tended to be higher than those in group-housed mice (Figure 5A). In the mice that ingested Arg at 3 mg/kg under the stressed condition, the expression of these genes was significantly suppressed (Figure 5A). In the prefrontal cortex, the expression levels of *Nr4a1*, *Arc*, and *Cyr61* between the control mice that were group-housed and confrontationally housed did not differ (Figure 5B). The expression levels of these genes were significantly suppressed in mice that ingested Arg under confrontational housing (Figure 5B).

#### 2.2.4. Effect of Arg Intake on Hba-a2 and Hbb-b2 Levels in the Brain

As *Hba-a2* and *Hbb-b2* are the most significantly upregulated genes in the hippocampus after Arg intake and are important factors in maintaining the function of neuronal mitochondria, we focused on these genes. qRT-PCR results indicate that *Hba-a2* levels in the hippocampus of confrontationally housed mice were similar to those of group-housed mice (Figure 5A); however, in the mice that ingested Arg, *Hba-a2* levels were significantly increased in both group-housed and confrontationally housed mice (Figure 5A). Similarly, in the prefrontal cortex, *Hba-a2* expression levels were significantly increased in mice that ingested Arg under group housing (Figure 5B). The levels of *Hbb-b2* expression in the hippocampus (Figure 5A) and prefrontal cortex (Figure 5B) were significantly increased in mice that ingested Arg under group housing and tended to increase in mice that ingested Arg under confrontational housing.

## 3. Discussion

In this study, we found that Arg exerts a remarkable anti-stress effect on the brain as a new function. A significant increase in oxidative damage was observed in the brain of stress-loaded SAMP10 mice, and these mice exhibited cognitive decline as well as a shortened lifespan as they aged. No lifespan-shortening effect due to confrontational housing was observed in WT mice such as ddY and C57BL/6 (data unpublished). Oxidative stress plays a crucial role in the aging process, particularly in cognitive dysfunctions [48]. Previous data suggested that dietary Arg supplementation ameliorated oxidative stress and that oral administration of Arg at a dose of 1.6 g/day for three months substantially reduced LPO levels in patients with senile dementia [49]. Our Arg dose administered to SAMP10 mice (3 mg/kg) was lower than the dose in the above report, but it supports that Arg has an inhibitory effect on LPO. 

Next, we looked at the molecular target of Arg in the brain to elucidate its mechanism in suppressing oxidative damage. The expression of several immediate-early genes (IEGs) such as *Nr4a1*, *Arc*, and *Cyr61* significantly increased in the hippocampus of stressed mice and was suppressed in mice that ingested Arg (Figure 5A). *Nr4a1* is associated with adrenal stress response and excessive neuronal excitotoxicity [37] and is known to be a potent pro-apoptotic molecule that induces nerve cell death [38,39]. Most importantly, Nr4a1 activated in response to oxidative stress has been reported to translocate from the nuclei to the mitochondria and induce mitochondrial damage and cell death [50]. Arc reportedly plays a critical role in the neuronal excitotoxicity mediated by glutamate receptor signaling. Moreover, an elevated mRNA and protein expression of Arc has been detected in rat cortical neurons via neurotoxic stimulation [40]. Cyr61 induction has been associated with neuronal cell death [45], and Cyr61 elevation has been reported to be associated with oxidative stress as it was markedly upregulated at both the gene and protein levels against reactive oxygen species induction in human dermal fibroblasts cells [51]. 

Arg incorporated into the brain has been shown to completely block glutamate-induced neuronal excitation in the ventromedial hypothalamus of rats [52]. Our data suggest that dietary Arg incorporated into the brain suppresses the stress-induced elevation of *Nr4a1*, *Arc*, and *Cyr61* in the hippocampus and that the suppression of these genes may be involved in preventing neuronal cell death through the regulation of excessive neuronal excitotoxicity and mitochondrial damage via the suppression of oxidative damage in the brain. 

Contrarily, *Hba-a2* and *Hbb-b2* expression levels were increased in the hippocampus and prefrontal cortex of mice that ingested Arg under both group and confrontational housing. As both Hba and Hbb are co-localized within the mitochondrion of neurons and are closely associated to maintain the neuronal mitochondrial function as well as survival of neurons [47], we speculate that Arg protects neurons by maintaining the neuronal mitochondrial function. An increase in the expression of Hba and Hbb reportedly has therapeutic effects against neurodegenerative disease, and increasing evidence suggests that a deficiency in these chains in the brain is associated with neurodegenerative disease [53,54]. Our results suggest that Arg may also be important in protecting the brain from neurodegenerative diseases such as Alzheimer’s disease. 

Arg revealed a similar protective effect to that of theanine on stress-induced shortened lifespan, cognitive decline, and depression in SAMP10 mice. We have shown that theanine significantly altered the gene expression of neuronal PAS domain protein 4 *(Npas4*) and lipocalin 2 (*Lcn2*) in the hippocampus of stressed SAMP10 mice [8]. Therefore, theanine and Arg suppress stress in different ways. 

In the present study, we demonstrated that orally administered Arg modulates psychosocial stress-induced gene expression in the hippocampus and prefrontal cortex of SAMP10 mice. Further study is necessary to elucidate how dietary Arg modulates the gene expression of *Hba-a2*, *Hbb-b2*, and IEGs (*Nr4a1*, *Arc*, and *Cyr61*) in the brain. It may be necessary to determine by immunofluorescence study that which cell types in the hippocampus and frontal cortex, namely, glial or neuron, is primarily reduce lipid peroxidation by Arg. In addition, the effect of Arg on mitochondrial morphology and function needs to be elucidated. 

## 4. Materials and Methods

### 4.1. Animals, Arg Preparation, and Housing Condition

Four-week-old male SAMP10/TaSlc (SAMP10) mice were purchased from Japan SLC Co., Ltd. (Shizuoka, Japan), and bred under conventional conditions in a temperature- and humidity-controlled room with 12/12 h light–dark cycle (light period, 08:00–20:00; temperature, 23 ± 1 °C; relative humidity, 55 ± 5%). Female mice were not used in this experiment because females are not as territorial as males. The mice were fed a normal diet (CE-2; Clea Co., Ltd., Tokyo, Japan). Arg (Wako Pure Chemical Co., Ltd., Osaka, Japan) was dissolved in water at 10 µg/mL. The volume of water containing Arg consumed by the mice was measured. Forty-eight mice were divided into four groups and observed to determine the long-term effects of Arg on their cognitive function and depression at nine months of age. Thereafter, the mice were fed continually to measure their lifespan. To study the effect of Arg on the brain, another group of 24 mice was used: 12 mice ingested Arg by drinking water ad libitum at 10 µg/mL (3 mg/kg) from one month of age. The remaining 12 mice ingested water as a control. Arg solution was freshly prepared twice a week. All experimental protocols were approved by the University of Shizuoka Laboratory Animal Care Advisory Committee (approval No. 166197, 5 April 2016) and were in accordance with the guidelines of the US National Institutes of Health for the care and use of laboratory animals. 

### 4.2. Housing Condition for Confrontation

**Figure 6:**
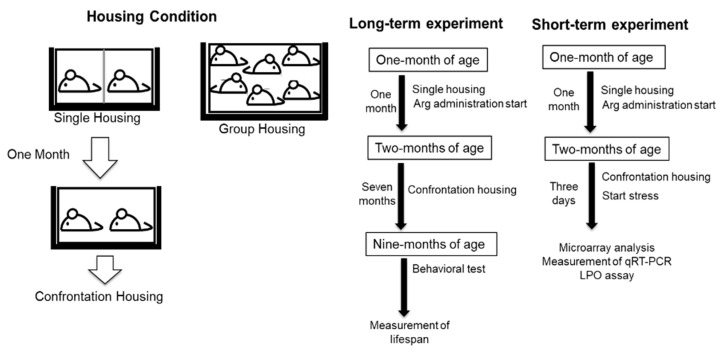
Two mice were housed in a cage with a stainless-steel partition (single housing) for one month. The partition was withdrawn, and mice were co-housed confrontationally in the same cage (confrontation housing) to load psychosocial stress. Group housing mice were kept with six in a cage. For the long-term experiment, after confrontationally housing mice for seven months, a behavioral test was performed at nine months of age; thereafter, their lifespan was measured. For the short-term experiment, after confrontationally housing mice for three days, microarray analysis, qRT-PCR, and LPO assay were performed.

To induce psychosocial stress in mice, confrontational housing was used, as previously described [1,2,4,8]. To build territoriality, two mice were housed for one month in a standard polycarbonate cage that is equally divided into two units by a stainless-steel partition. Next, the partition was removed, and the mice were co-housed confrontationally to induce psychosocial stress (Figure 6). Group housing was used as a model of the low-stressed condition. Two experiments in a linear diagram are shown including each experimental step for long term and short term (Figure 6). 

### 4.3. Memory Acquisition Test 

To measure the learning ability of mice, a step-through passive avoidance task was tested on 9-month-old mice, as previously described [2]. Briefly, when a mouse enters a dark chamber from a light chamber, the door in the chamber is closed and an electric foot-shock is delivered at 50 µA for 1 s (SGS-003, Muromachi Kikai. Co., Ltd., Tokyo, Japan). The acquisition of the avoidance response was considered successful if the mouse remained in the light chamber for 300 s. The trial was repeated until the mouse satisfied the acquisition criterion within five trials. For each trial, the time spent by the mice in the light chamber was subtracted from 300 s; the results from the successive trials were summed up for each mouse to determine the time required for learning (“learning time”).

### 4.4. Measurement of Immobility in the Tail Suspension Test

To examine behavioral depression, mice were individually suspended by their tails at a height of 30 cm using a clip for tail suspension (MSC2007, YTS Yamashita Giken, Tokushima, Japan). The duration of immobility was recorded for 15 min, as previously described [1]. The mice were considered immobile only when they were both passively hanging and completely motionless. The duration during which the mice were immobile was measured.

### 4.5. Measurement of Oxidative Damage in the Brain

Mice that were confrontationally housed for three days after being singly housed for one month were used for the quantification of LPO. LPO in the brain of SAMP10 mice was measured using a lipid hydroperoxide assay kit (Cayman Chemical Company, Ann Arbor, MI, USA) according to the manufacturer’s instructions. Briefly, approximately 50 mg of the cerebral cortex was homogenized in 500 µL of HPLC-grade water. An equal volume of methanol solution saturated with Extract R^®^ was added following the addition of 1 mL chloroform–methanol (2:1, *v*/*v*) solvent. After centrifugation at 1500× *g* for 5 min, the bottom layer of the chloroform was collected, and the lipid hydroperoxide content was measured via redox reactions with ferrous ions. The resulting ferric ions produced from the reaction of hydroperoxide with the ferrous ions were detected using thiocyanate ion as the chromogen. The absorbance of each sample at 500 nm was obtained (*n* = 6/group).

### 4.6. Measurement of DNA Microarray and qRT-PCR

The mice that were confrontationally housed for three days after being singly housed for one month were used for DNA microarray analysis. The mice were provided 10 µg/mL (3 mg/kg) Arg-water ad libitum. RNeasy Mini Kit (NucleoSpin^®^ RNA, 740955, Takara Bio Inc., Shiga, Japan) was used to extract total RNA from the hippocampus. To synthesize biotinylated cRNA, total RNA was processed using One-Cycle Target Labeling and Control Reagents (Affymetrix, Santa Clara, CA, USA) and hybridized to a Total RNA Mouse Gene 2.0 ST Array (Affymetrix) using three biological repeats per group. The significance of Arg intake was statistically tested using two-way ANOVA at *p* < 0.001 [55,56].

For the measurement of qRT-PCR, group-housed same-aged mice were used as the reference. Total RNA was isolated from the homogenized hippocampus and prefrontal cortex as described above. cDNA was prepared from the obtained RNA using PrimeScript^®^ RT Master Mix (RR036A, Takara Bio Inc.). qRT-PCR analysis was performed using PowerUp^™^ SYBR^™^ Green Master Mix (A25742, Applied Biosystems Japan Ltd., Tokyo, Japan) and automated sequence detection systems (StepOne, Applied Biosystems Japan Ltd., Tokyo, Japan). Previously validated primers for *Nr4a1*, *Arc*, *Cyr61*, *Hba-a2*, and *Hbb-b2* [37,57,58,59,60] (Table 2) were used to quantify their relative gene expression. β-actin was used as the internal control.

### 4.7. Statistical Analysis

Statistical data are presented as the mean ± standard error of the mean. Statistical analysis was performed using one-way ANOVA followed by Tukey–Kramer’s honest significant difference method for cognition activity and a tail suspension test. Fisher’s least significant differences were used for qRT-PCR and the LPO assay. After calculating survival rates using the Kaplan–Meier method, the difference in survival rate was tested using the log-rank test. *p* < 0.05 was considered statistically significant.

## 5. Conclusions

The present study revealed that the daily intake of Arg at a dose of 3 mg/kg suppressed cognitive decline and depression-like behavior and counteracted shortened lifespan in chronic stress-loaded SAMP10 mice. We speculate that Arg reduces stress by suppressing oxidative damage in the brain, resulting in slowed aging. The suppression of *Nr4a1*, *Arc*, and *Cyr61*, which are associated with oxidative damage and nerve cell death, and an increase in *Hba-a2* and *Hbb-b2*, which protect neuronal mitochondrial dysfunction, were observed in mice that ingested Arg. Arg may be a potential candidate for the suppression of the deleterious effects of chronic psychosocial stress.

## Figures and Tables

**Table 1:** Top 10 significantly downregulated and upregulated genes in the hippocampus of mice that ingested Arg under confrontational housing.

	Symbol	Full Name	ΔZ	*p*
**Downregulated**	Nr4a1	Nuclear receptor subfamily 4, group A, member 1	−0.2726	3.51 × 10^−10^
	Arc	Activity regulated cytoskeleton-associated protein	−0.2345	2.51 × 10^−7^
	Olfr1384	Olfactory receptor 1384	−0.2446	0.00702
	Cryba1	Crystallin, beta A1	−0.2270	0.00280
	Ms4a6d	Membrane-spanning 4-domains, subfamily A, member 6D	−0.2371	0.00674
	Mid1	Midline 1	−0.2399	2.93 × 10^−9^
	Cyr61	Cysteine rich protein 61	−0.2591	8.73 × 10^−10^
	Prss2	Protease, serine 2	−0.2197	0.00620
	H2-Q6	Histocompatibility 2, Q region locus 6	−0.2550	0.00548
	Mir1983	MicroRNA 1983	−0.1860	0.00375
**Upregulated**	Mela	Melanoma antigen	0.3509	1.42 × 10^−5^
	Olfr2	Olfactory receptor 2	0.3302	0.00402
	Slitrk6	SLIT and NTRK-like family, member 6	0.1712	0.00023
	Hbb-b2	Hemoglobin, beta adult minor chain	0.3780	3.55 × 10^−8^
	Itih2	Inter-alpha trypsin inhibitor, heavy chain 2	0.2867	7.42 × 10^−6^
	Olfr535	Olfactory receptor 535	0.3544	0.00262
	Zic1	Zinc finger protein of the cerebellum 1	0.0703	4.38 × 10^−9^
	LOC666331	Uncharacterized LOC666331	0.2908	0.00262
	Hba-a2	Hemoglobin alpha, adult chain 2	0.3284	6.38 × 10^−17^
	Tcf712	Transcription factor 7 like 2, T cell specific, HMG box	0.1181	1.27 × 10^−11^

ΔZ = expression level (confrontation Arg-confrontation control).

**Table 2:** Primer sequences for qRT-PCR.

Gene	Forward Sequence (5′–3′)	Reverse Sequence (5′–3′)	Ref.
*Nr4a1*	CTGCCTTCCTGGAACTCTTCA	CGGGTTTAGATCGGTATGCC	[37]
*Arc*	ACGATCTGGCTTCCTCATTCTGCT	AGGTTCCCTCAGCATCTCTGCTTT	[57]
*Cyr61*	CCCCCGGCTGGTGAAAGTC	ATGGGCGTGCAGAGGGTTGAAAAG	[58]
*Hba-a2*	GAAGCCCTGGAAAGGATGTT	GCCGTGGCTTACATCAAAGT	[59]
*Hbb-b2*	CACCTGACTGATGCTGAGAAGT	CCCTTGAGGTTGTCCAGGTTT	[60]

## Data Availability

The data presented in this study are available on request from the corresponding author.

## Abbreviations

Arg	Arginine
Arc	Activity regulated cytoskeletal-associated protein
Cyr61	Cysteine rich protein 61
Hba-a2	Hemoglobin alpha, adult chain 2
Hbb-b2	Hemoglobin, beta adult minor chain
IEGs	Immediate-early genes
LPO	Lipid peroxidation
MST	Median survival time
Nr4a1	Nuclear receptor subfamily 4, group A, member 1
qRT-PCR	Quantitative real-time reverse transcription polymerase chain reaction
SAMP10	Senescence-accelerated mouse prone 10
